# Prospective study to optimize the health of patients with TIAS (transient ischemic attack) and stroke admitted to the Hamad General Hospital

**DOI:** 10.1097/MD.0000000000020694

**Published:** 2020-07-10

**Authors:** Yahia Z. Imam, Mouhand F.H. Mohamed, Mohamed S. Abdelmoneim, Mark Santos, Nima Alkhawad, Abdul Salam, Numan Amir, Maher Saqqur, Ahmad Muhammad, Ahmed Elsoutohy, Saadat Kamran, Naveed Akhtar, Abdul Salim Kiliyanni, Ahmed Own, Dirk Deleu, Ashfaq Shuaib

**Affiliations:** a Neuroscience Institute, Hamad Medical Corporation; b Weill Cornell Medicine-Qatar; c Internal Medicine Department, Hamad General Hospital, Hamad Medical Corporation; d Radiology Department, Hamad General Hospital, Hamad Medical Corporation, Doha, Qatar; e Division of Neurology, Department of Medicine, University of Alberta, Edmonton, Canada.

**Keywords:** cerebrovascular disease, diabetes, hypertension, pharmacist, stroke

## Abstract

**Introduction:**

Recurrent ischemic strokes (IS) make up to one-third of all strokes. Nine out of 10 strokes are due to modifiable risk factors. Thus, it seems that standard management strategies of modifiable risk factors are yet to improve. Hence, we planned a randomized controlled trial assessing nurses or pharmacists-led aggressive control of comorbidities and their prognostic impact on IS and transient ischemic attacks (TIA).

**Methods/design:**

Prospective study to optimize the health of patients with TIAs and stroke admitted to the Hamad General Hospital (PROMOTE HEALTH) is an assessor-blinded, open-label, randomized, two-arm, controlled trial. Eligible patients have IS or TIA, and an additional modifiable risk factor (Hypertension or dyslipidemia) attending the stroke ward or clinic at the Weill Cornell-affiliated Hamad General Hospital. Stroke specialists will offer the control group the currently practiced best risk factor management strategies. Whereas, in the intervention arm, with the assistance of a nurse and a pharmacist, we will make aggressive attempts to meet targets of defined risk factors. The primary outcomes are the mean difference in blood pressure (BP) and low-density lipoprotein. Whereas myocardial infarction, recurrent stroke events, and mortality serve as the study's secondary outcomes. We require 200 patients per study arm to achieve a power of 80% and an alpha level of <0.05. The Medical Research Center and the Institutional Review Board have approved the study, and it was prospectively registered in a trial registry.

**Discussion:**

A significant proportion of strokes are due to modifiable preventable risk factors. Despite having the right preventive strategies aimed at mitigating these risk factors, a sizeable proportion of strokes are due to recurring events. This prompted the medical community to evaluate aggressive means of addressing these risk factors. The nurse or pharmacist-led management of comorbidities has been proven to be of value in the management of diabetes and hypertension. It will be of value to demonstrate the effectiveness of utilizing this additional task force in aggressively managing IS or TIA patients with an overarching goal of improving their prognosis. If our intervention proves to be efficacious, this would have a substantial impact on the current stroke practices and guidelines. Additionally, it will invite further research in the area.

**Clinical trial registration:**

Clinicaltrials.gov NCT02868723, last updated on September 2018

## 1 Introduction

Cardiovascular disease accounts for more than 50% of deaths in the adult population, and its impact on society is immense.^[1]^ Cerebrovascular disease is a significant contributor to the problem, and its incidence is rising in the Gulf area population and around the globe. Evidence from multiple sources has shown that with aggressive risk factor management, stroke, especially recurrent stroke, is preventable.^[2]^ While there are recent studies from Qatar and the middle east on stroke subtypes, risk factors, hospital admission rates, and early prognosis, comprehensive research on the effects of prevention on disease progress or regression is urgently needed.^[3,4]^

We hypothesize that aggressive management of vascular risk factors to “recommended target levels” will lead to better vascular health. Compared to current practice, a comprehensive and coordinated approach at preventive measures will lead to more patients with better control of blood pressure (BP) and lipid levels. Improved risk factor management will result in slowing of atherosclerosis and its downstream effects, which will be measurable on sophisticated blood and imaging testing. Clinically this will translate into fewer hospital readmissions. Hence, we planned a randomized controlled trial to determine if a comprehensive and coordinated approach (led by nurses and pharmacists) at risk factor management will lead to more patients with better control of BP and lipid levels. Moreover, it will determine if better control of BP and lipid levels results in the slowing of plaque progression as measured on 3D Carotid Doppler imaging studies. Additionally, we will examine the effect of this approach on the mortality, stroke recurrence, and myocardial infarction incidence.

## 2 Methods/design

### 2.1 Study design and settings

This will be an open-label, two-arms, parallel, prospective randomized controlled study with a blinded assessment. We will recruit eligible patients admitted to the Weill Cornell affiliated Hamad General Hospital (HGH) or attending the Stroke Prevention Clinic with a diagnosis of ischemic stroke (IS) or transit ischemic attacks (TIAs). Potentially eligible patients will be invited to participate in our trial. If they agreed, they will be screened for eligibility and consented before being allocated to a study arm. The study is approved by the institutional review board (IRB) and the medical research center (MRC) of HGH. Additionally, it received an internal grant from the MRC (IRGC-2-NI-059). The trial is registered at clinicaltrials.gov (NCT02868723).

### 2.2 Setting and participants

This is a single-center study. All patients with a first TIA or IS would be approached for participation in the study. Patients who, in addition, also have a history of hypertension or increased low-density lipoprotein (LDL) blood levels would be enrolled from the HGH inpatient wards and the Stroke Prevention/rapid access TIA Clinic. All patients will be screened for eligibility to participate in our study based on the following criteria:

**Inclusion criteria**

1. Ischemic stroke or TIA including ocular stroke such as Amaurosis Fugax, within the past year (not intracranial hemorrhage or due to trauma, malignancy or cardio-embolic related to structural heart disease)
2. Meets at least one of the following three criteria:
  - Systolic BP >140 mm Hg but <200 mm Hg average of 3 readings taken within 24 hours of admission and if based on clinic visits, then an average of three readings at least separated by 5 minutes-interval.
  - Fasting LDL cholesterol >2.0 (measured within the previous 6 months)
  - Total: HDL cholesterol ratio > 4.0 (measured within the previous 6 months)
3. Willing to participate (by signing a consent form) and willing to return for study-related scheduled follow up visits for 1 year from the time of enrollment into the study.
4. Age 18 to 90 years.

**Exclusion criteria**

1. Participation in a concurrent interventional trial related to stroke or vascular disease.
2. Any condition, including foreshortened life-expectancy, or severe comorbidities that would preclude treatment benefit or 12-month follow up.
3. Institutionalized patients in a long-term care facility.
4. Receiving maximal therapies for the inclusion comorbidity (3 maximal doses antihypertensives or maximal doses of statins.
5. Computed tomography or magnetic resonance imaging shows evidence of primary intracranial hemorrhage or neoplasm.
6. Active coronary artery disease.
7. Severe renal disease on hemodialysis (HD) or severe hepatic dysfunction
8. Decline of participation or inability to participate in our trial (Not able to follow in our center, or with cognition, mobility, or language barriers etc.).
9. A severe systemic illness that will not allow for the patient to complete the 1-year trial.
10. History of intolerance to statins or commonly used anti-hypertensive medications (intolerance/contraindication to statin (if elevated LDL-c is the inclusion criteria)
11. Unable to tolerate antiplatelet agents.

### 2.3 Randomization, allocation concealment, and blinding

An expert trial investigator will screen the patients for eligibility and consent for enrollment. Within 48 hours of screening and consenting, patients will be randomized to one of the 2 study arms. Computer-generated randomization will be performed with concealment of allocation. Random variable sized-blocks^[2–4]^ will be used to allocate patients to either of the study arms. The trial is open label; however, outcome assessors and data analysts will be blinded to the patient allocation.

### 2.4 Interventions

**Figure 1:**
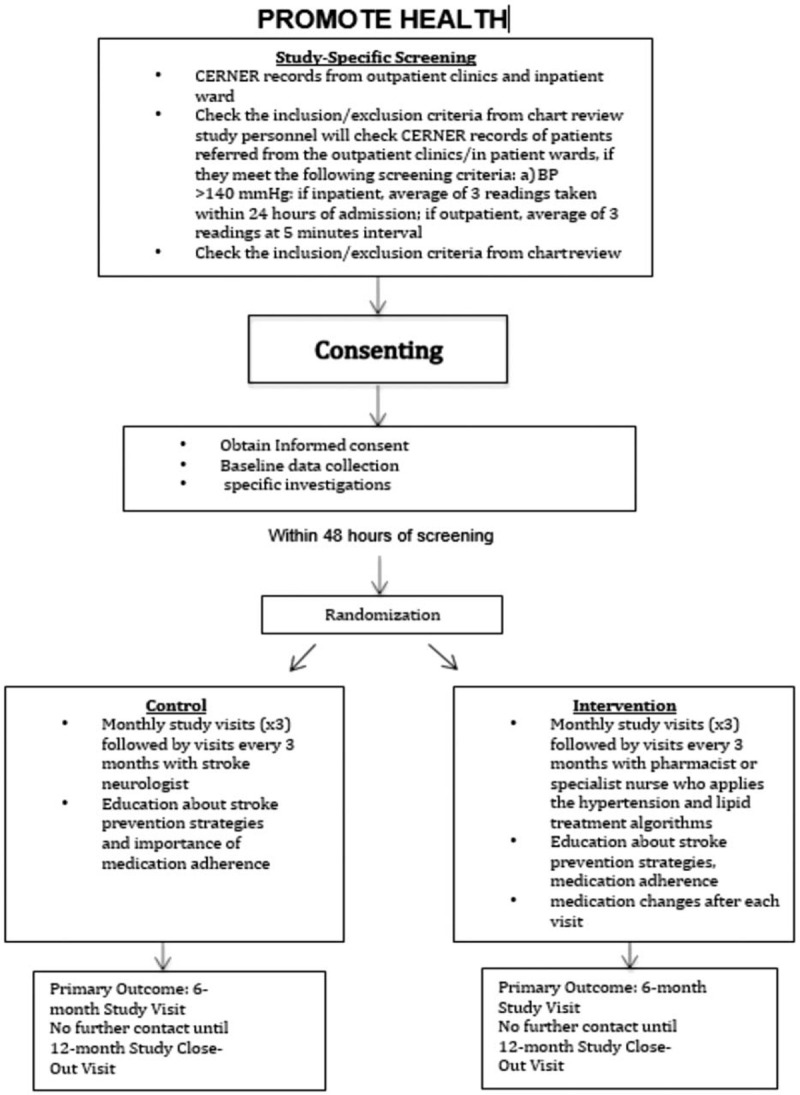
Flow diagram depicting the study procedures.

After consent and initial evaluation and investigations, patients in the standard of care arm will have their risk factors managed according to the current protocols in place at the hospital in the prevention of recurrent stroke (Fig. 1). Whereas patients in the aggressive, coordinated management (intervention) arm will be offered a comprehensive review on mechanisms of stroke/vascular disease. Every attempt will be made to bring their risk factors to optimal control based on internationally accepted guidelines with assistance from a specially trained pharmacist and nurse practitioner. Both arms will be followed (initially every month for three months and then every three months) for one year. All stroke specialists will run the standard arm guided by HGH local protocol. Imaging studies and other tests completed at the start of the study will be repeated at the exit from the study one year following randomization. Smoking cessation programs will be offered to patients who continue to smoke, and regular exercise would be strongly encouraged. The Framingham Risk Score report cards would be discussed on each visit, which will include the status of risk factor management.

### 2.5 Data collection

The study will be based in Hamad Hospital, Doha, Qatar where patients will be recruited from within the stroke program. The case record forms (CRFs) will be filled by the study investigators. If a change in a CRF is deemed necessary, a single line over what needs to be amended, to maintain it legible for reading. Then the investigator needs to countersign this addendum. CRFs will be collected be kept under lock and key. Data entries will be monitored daily, and data backed up electronically, the access to the electronic database will be limited to the PI.

### 2.6 Primary outcomes

The mean difference in the BP and LDL are the study's two primary outcomes.

### 2.7 Secondary outcomes

Recurrent events of stroke, myocardial infarction (MI), or death. Additionally, carotid plaque progression as measured on 3D Carotid Doppler imaging studies

### 2.8 Statistical consideration

#### 2.8.1 Sample size

Based on an estimated clinically meaningful BP difference of 10% between the two study groups. We will require 200 patients in each group (alpha error set at 0.05 and beta error at 0.20, power 80%, and an estimated dropout rate of 20%).

#### 2.8.2 Analysis set

Intention to treat analysis will be utilized for all the study outcomes.

#### 2.8.3 Statistical analysis

We will utilize descriptive statistics to summarize the baseline data, clinical, and laboratory characteristics of our study population. Continuous variables will be summarized using means (standard deviation) and medians (interquartile range). We will utilize the ANOVA or Mann Whitney test to compare continuous data (based on data normality). Categorical variables will be compared using the Chi-squared tests. Additionally, uni and multivariate regression will be attempted in our study. All analyses will be performed using SPSS version 22.

### 2.9 Quality control and trial management

The trial is approved by IRB and MRC and is following all ethical guidance (ethical Declaration of Helsinki). The study investigators will meet regularly to ensure optimal trial conduct. Additionally, a data and safety monitoring board (DSMB) will oversee the whole trial conduct with ongoing auditing and monitoring of the trial procedures. The DSMB will comprise nominated members from the Medical Research Center. All serious adverse events (SAE) will be brought to the attention of the trial team momentarily. The PI and the project manager, in turn, will update the DSMB.

Investigators will report all serious adverse events (SAE). The SAE reporting period includes the entire treatment duration and will focus mainly on medication-related side effects. Additional AEs of relevance to our study are prolonged uncontrolled hypertension, poor lipid control, and side-effects/discontinuance of medications. The DSMB will also monitor these. An interim analysis was deemed unnecessary, and thus, has not been planned in our trial (Discussed with IRB).

## 3 Discussion

Stroke is the most common neurological severe diagnosis requiring hospital admission. The management and the prognosis of this entity are improving, yet it remains to be a major cause of morbidity and mortality. A report from 2017 demonstrated that stroke was the fifth leading cause of mortality in the united states.^[1]^ It is estimated that 9 out of 10 strokes are resulting from modifiable factors.^[2]^ The most common modifiable factors are hypertension, diabetes, dyslipidemia, obesity, and smoking.^[1,2]^

Prompt identification and treatment, addressing the modifiable risk factors, constitute the best stroke management strategy. However, addressing these secondary risk factors are not always successful, with recurrent strokes constituting up to a third of all new strokes.^[5]^ Another evidence suggests that there is a striking lack of awareness of the condition in the general population (less than 30% aware of the entity), and less than a third of the population is aware that hypertension and diabetes are important risk factors.^[3]^ This, in part, may explain why such strategies are ineffective. Stroke leaves the patients with considerable disability. In patients with large artery atherosclerosis, more than 50% will have moderate to severe disability 90 days after the stroke onset.^[6,4]^ Our experience over 3 decades has shown that this cohort of patients (patients with an acute IS or TIA) are a very receptive and committed to the offered medical advice.

The earlier mentioned factors were the driving force behind the design of this trial. The primary focus of the proposed PROMOTE-HEALTH study is on improvement in risk factor management in patients presenting with a stroke or TIA. A positive study that shows that we can indeed improve risk factor management by an aggressive approach will have significant implications for health care. Especially if we can show that better risk factor management reduces the recurrence or slows the progression of the disease as measured by the parameters defined in our primary and secondary outcomes. We have carefully selected the targets for our interventions intended to be used in our study (Blood pressure and LDL cholesterol control). There is strong evidence for preventing vascular disease with appropriate management of these prevalent modifiable risk factors in this cohort of patients.^[1,2]^ Our selection of the nurses and pharmacists to deliver our intervention (aggressively controlling the modifiable risk factors was backed by evidence from other diseases. Data from 3 systematic reviews and meta-analyses (2007, 2010) demonstrated that nurse or pharmacist-led comorbidities management resulted in a better diabetes and hypertension control, respectively.^[7–9]^ Whether these benefits extend to other chronic vascular conditions is not known and is the premise of the PROMOTE-HEALTH study.

Findings from our study will carry significant weight in the future management of stroke patients locally and globally. We anticipate finishing the study by 2021. The study results will be disseminated through presentations at local, regional, and international conferences. In addition, we will publish the final results in a high-impact medical or neurology Journal.

## Author contributions

YZI, AsS has conceived the idea of this trial and is the PI of this study. YZI/AsS is responsible for the design of the trial and AS statistical analysis. YZI & DD planned the full protocol of the study. YZI wrote and revised the protocol that was submitted to and approved by MRC. MFHM adapted the protocol for publication. The adapted version was critically revised by YZI. All authors revised the final version critically and approved it for publication. Additionally, all co-authors will be responsible for the overall conduct of the trial and final manuscript writing.
